# Körperliche Funktionsfähigkeit und Gebrechlichkeit (Frailty) im hohen Alter in Deutschland

**DOI:** 10.1007/s00103-026-04280-0

**Published:** 2026-08-11

**Authors:** Sonja Nowossadeck, Svenja M. Spuling, Stefan Stuth, Judith Fuchs

**Affiliations:** 1 Deutsches Zentrum für Altersfragen (DZA), Manfred-von-Richthofen-Straße 2, 12101 Berlin, Deutschland; 2 Robert Koch-Institut, Nordufer 20, 13353 Berlin, Deutschland

**Keywords:** Hochaltrige, Körperliche Funktionsfähigkeit, Gebrechlichkeit, Alltagsfunktionalität, Funktionale Einschränkungen, Oldest old, Physical functioning, Frailty, Everyday functioning, Functional limitations

## Abstract

**Einleitung:**

Körperliche Funktionsfähigkeit ist zentral für Autonomie und Teilhabe im hohen Alter. Ziel der Arbeit ist es, funktionale Einschränkungen bei Hochaltrigen in Deutschland anhand eines globalen Indikators (Global Activity Limitation Indicator - GALI) und bereichsspezifischer Indikatoren (Alltagsfunktionalität, Mobilität, Sensorik, Harninkontinenz) zu beschreiben. Ergänzend wird Gebrechlichkeit (Frailty) als Risikofaktor untersucht.

**Methoden:**

Die Analysen basieren auf Daten des Deutschen Alterssurveys (DEAS) 2023 (*n* = 938) und der Studie Gesundheit 65+ 2021/2022 (*n* = 2028) für Personen ab 80 Jahren in Privathaushalten. Der GALI wird datensatzübergreifend, weitere Indikatoren datensatzspezifisch ausgewertet. Die Ergebnisse werden nach Geschlecht und Einkommen differenziert.

**Ergebnisse:**

Etwa ein Viertel der Hochaltrigen ist stark funktional eingeschränkt; dies zeigt sich konsistent in beiden Datensätzen. Bei der Alltagsfunktionalität zeigen Männer signifikant bessere Werte als Frauen. Rund ein Drittel ist beim Gehen von mehr als einem Kilometer und knapp ein Sechstel beim Treppensteigen stark eingeschränkt. Schwere sensorische Einschränkungen betreffen 10 % beim Sehen und 27 % beim Hören. Harninkontinenz berichten 40 % der Hochaltrigen, Frauen häufiger als Männer. Knapp die Hälfte ist nach den gewählten Kriterien robust, 43 % „pre-frail“ und 8,6 % „frail“. Unterschiede zwischen Einkommensgruppen zeigen sich nur teilweise und nicht konsistent.

**Diskussion:**

Funktionale Einschränkungen sind bei Hochaltrigen in Privathaushalten weitverbreitet, insbesondere bei Frauen und teils bei niedrigen Einkommen. Sensorische Defizite, Harninkontinenz und Frailty sind häufig und haben eine hohe Versorgungsrelevanz. Prävention und Versorgung sollten früh, geschlechter- und soziallagenbezogen ansetzen.

**Zusatzmaterial online:**

Zusätzliche Informationen sind in der Online-Version dieses Artikels (https://doi.org/10.1007/s00103-026-04280-0) enthalten.

## Abstract (en)

**Introduction:**

Physical functional capacity is central to autonomy and participation in very old age. The aim of this study is to describe functional limitations among the oldest old in Germany using a global indicator (Global Activity Limitation Indicator - GALI) and domain-specific indicators (activities of daily living, mobility, sensory function, urinary incontinence). In addition, frailty is examined as a risk factor.

**Methods:**

The analyses are based on data from the 2023 German Ageing Survey (DEAS) (*n* = 938) and the 2021/22 Health 65+ study (*n* = 2028) for persons aged 80 and older living in private households. GALI is analysed across datasets, while further indicators are analysed separately for each dataset. Results are stratified by gender and income.

**Results:**

About one quarter of the oldest old have severe functional limitations; this finding is consistent across both datasets. In daily living activities, men show significantly better scores than women. Around one third are severely limited in walking more than one kilometre, and just under one sixth are severely limited in climbing stairs. Severe sensory limitations affect 10% in vision and 27% in hearing. Urinary incontinence is reported by 40% of the oldest old, more often by women than by men. According to the selected criteria, almost half are robust, 43% are pre-frail and 8.6% are frail. Differences between income groups are only partly evident and are not consistent.

**Discussion:**

Functional limitations are widespread among the oldest old living in private households, particularly among women and, in some cases, among those with low income. Sensory impairments, urinary incontinence and frailty are common and highly relevant for health and social care. Prevention and care should start early and take gender and socioeconomic position into account.

## Einleitung

Unter Funktionsfähigkeit versteht die Weltgesundheitsorganisation (WHO) die gesundheitsbezogenen Attribute, die es Menschen ermöglichen, ihr Leben und ihre Aktivitäten entsprechend ihren individuellen Werten und Zielen zu gestalten [1]. Eine gute körperliche Funktionsfähigkeit ermöglicht es, den Alltag eigenständig zu bewältigen, mobil zu bleiben und soziale Beziehungen aufrechtzuerhalten, und ist damit eng mit Lebensqualität, Autonomie und sozialer Integration verknüpft. Einschränkungen gehen dagegen häufig mit Autonomieverlust, erhöhter Unterstützungsbedürftigkeit und einem erhöhten Risiko sozialer Isolation einher [2, 3]. Mit Frailty (Gebrechlichkeit) liegt seit mehreren Jahrzehnten ein Konzept vor, welches über die körperliche Funktionsfähigkeit hinaus das gleichzeitige Auftreten verschiedener, z. T. krankheitsbedingter Einschränkungen verwendet, die ältere Menschen (gesundheitlich) weniger belastbar und damit anfälliger für Erkrankungen, Behinderungen oder Stürze machen [4].

Mit dem Erreichen der Hochaltrigkeit – in der Forschung meist ab dem 80. Lebensjahr definiert – steigt das Risiko für funktionale Einschränkungen, Mobilitätsverluste und Frailty deutlich. Dies kann den Alltag erschweren und zu einem erhöhten Unterstützungs- oder Pflegebedarf führen. Zugleich wird sich der Anteil der Hochaltrigen an der Bevölkerung in den nächsten Jahrzehnten fast verdoppeln [5]. Damit wird die differenzierte Beschreibung funktionaler Kapazitäten und Defizite dieser Bevölkerungsgruppe wichtiger für Prävention und Versorgung. Folgende Dimensionen der körperlichen Funktionsfähigkeit bei Hochaltrigen werden in diesem Artikel untersucht: die globale Funktionalität (GALI), die bereichsspezifische Funktionalität (Alltagsfunktionalität, Mobilität, sensorische Funktionalität, Kontinenzfunktion) sowie Gebrechlichkeit (Frailty) als Risikofaktor.

### Globale und bereichsspezifische körperliche Funktionalität

Der *Global Activity Limitation Indicator (GALI)* zur Erfassung langfristiger globaler Einschränkungen der körperlichen Funktionsfähigkeit ist international etabliert, unter anderem in der europäischen Gesundheitsberichterstattung [6, 7]. Der GALI erfasst, ob Personen aufgrund eines gesundheitlichen Problems seit mindestens 6 Monaten in ihren üblichen Aktivitäten eingeschränkt sind.

Neben diesem globalen Indikator wird die körperliche Funktionsfähigkeit auch nach Funktionsbereichen differenziert: Ein zentraler Bereich ist die *Alltagsfunktionalität*, die sich in bevölkerungsbezogenen Studien über die Physical-Functioning-Skala (PF) des SF-36 abbilden lässt [8, 9]. Der SF-36-PF erfasst Einschränkungen bei grundlegenden alltagsrelevanten körperlichen Aktivitäten. Die *Mobilität* stellt einen Teilbereich dieser Alltagsfunktionalität dar. Sie ist Voraussetzung für außerhäusliche Aktivitäten und soziale Kontakte. Einschränkungen der Mobilität sind mit erhöhter Morbidität, Sturzrisiko, sozialer Isolation und Pflegebedürftigkeit assoziiert und gelten als zentraler Marker funktionaler Vulnerabilität im hohen Alter [10].

Neben der motorischen Dimension kommt der *sensorischen Funktionalität* – insbesondere Sehen und Hören – im hohen Alter große Bedeutung zu. Sensorische Einschränkungen sind weitverbreitet und betreffen grundlegende Prozesse der Informationsaufnahme, Orientierung und Kommunikation. Die WHO hebt hervor, dass sie erhebliche Auswirkungen auf Selbstständigkeit, soziale Interaktion und Lebensqualität haben können [3, 11]. Sensorische Funktionalität ist damit eine grundlegende Voraussetzung für Autonomie und Teilhabe im sehr hohen Alter.

Eine weitere, häufig unterschätzte, aber hochrelevante Domäne ist die Kontinenzfunktion, also die Fähigkeit des Körpers, Harn oder Stuhl willkürlich zurückzuhalten. Die im Artikel betrachtete *Harninkontinenz* ist im hohen Alter verbreitet. Sie wirkt unmittelbar in Alltagsroutinen, Mobilität und soziale Aktivitäten hinein. Systematische Übersichten zeigen, dass Harninkontinenz mit einer verminderten gesundheitsbezogenen Lebensqualität assoziiert ist [12].

### Gebrechlichkeit (Frailty) als Risikofaktor für körperliche Funktionalität

Im Gegensatz zu den Indikatoren körperlicher Funktionsfähigkeit, die bestehende Defizite und Einschränkungen abbilden, erfasst *Frailty* die altersbedingte Vulnerabilität und damit das Risiko funktionaler Verschlechterungen. Frailty ist ein klinisches Syndrom, das durch verminderte physiologische Reserven und eine erhöhte Anfälligkeit gegenüber Stressoren gekennzeichnet ist [13]. Menschen mit Frailty oder Vorstufen davon weisen eine besonders fragile funktionale Gesundheit auf und haben zudem ein erhöhtes Risiko für Stürze, Krankenhausaufenthalte, Behinderungen und Sterblichkeit [14–16]. Zudem ist ihr Risiko für Pflegebedürftigkeit erhöht [17, 18]. In der Forschung wird Frailty nach unterschiedlichen Konzepten definiert und operationalisiert. Im vorliegenden Artikel wird das phänotypische Konzept nach Fried [13] zugrunde gelegt.

### Gruppenunterschiede in körperlicher Funktionalität und Frailty

Im hohen Alter steigt die Wahrscheinlichkeit funktionaler Einschränkungen deutlich an, insbesondere wenn physiologische Reserven abnehmen und chronische Erkrankungen häufiger werden [3, 13]. Auch die Prävalenz des Risikofaktors Frailty und die Übergänge von robusten zu frailen Zuständen sind insbesondere in den höchsten Altersgruppen zu sehen [13, 19].

Darüber hinaus zeigen zahlreiche Studien Geschlechterunterschiede in der körperlichen Funktionsfähigkeit mit häufigeren Einschränkungen bei Frauen im Vergleich zu Männern, obwohl Frauen eine höhere Lebenserwartung haben – ein Befund, der häufig als „health-survival paradox“ beschrieben wird [20, 21]. Dieser Unterschied gilt auch für Frailty [22, 23]. Daneben sind soziale Gradienten auffällig: Ältere Menschen mit niedrigem Einkommen bzw. sozioökonomischem Status berichten häufiger funktionale Einschränkungen als sozial besser gestellte Gruppen [24] und sind häufiger von Frailty betroffen [25, 26].

Der vorliegende Beitrag verfolgt das Ziel, das Ausmaß funktionaler Einschränkungen und des Risikofaktors Frailty bei hochaltrigen Menschen in Deutschland differenziert zu beschreiben. Dabei versteht sich die Arbeit als deskriptive Darstellung ausgewählter Dimensionen körperlicher Funktionsfähigkeit. Die einbezogenen Indikatoren stammen aus zwei komplementären bevölkerungsbezogenen Datensätzen und bilden unterschiedliche Funktionsdomänen ab. Sie werden daher nicht zu einem Gesamtmaß integriert, sondern beschreiben einander ergänzend zentrale, für Selbstständigkeit und Teilhabe relevante Funktionsbereiche. Die Befunde berücksichtigen Geschlechts- und Einkommensunterschiede, um eine empirische Einordnung der körperlichen Funktionsfähigkeit im hohen Alter zu ermöglichen. Der Artikel untersucht folgende Forschungsfragen:

- Wie gut ist die globale Funktionalität der Hochaltrigen insgesamt und welches Ausmaß haben funktionale Einschränkungen in den Teilbereichen Alltagsfunktionalität, Mobilität, sensorische Funktionalität und Kontinenzfunktion?
- Wie weit sind die Risikofaktoren Frailty und Pre-Frailty in der hochaltrigen Bevölkerung verbreitet?
- Welche Geschlechts- und Einkommensunterschiede sind in der globalen und bereichsspezifischen Funktionalität sowie beim Frailty-Syndrom Hochaltriger zu beobachten?

## Methoden

### Datensätze und Studienpopulationen

Im Beitrag werden zwei unabhängige Datensätze analysiert: der Deutsche Alterssurvey (DEAS) und die Studie „Gesundheit 65+“.

Der DEAS ist eine repräsentative Quer- und Längsschnittstudie des Deutschen Zentrums für Altersfragen (DZA), die seit 1996 regelmäßig Frauen und Männer ab 40 Jahren in einer nach Alter, Geschlecht und Region geschichteten Einwohnermeldeamtsstichprobe befragt [27–29]. Die Daten des DEAS sind repräsentativ für die in Privathaushalten lebende Wohnbevölkerung Deutschlands in der zweiten Lebenshälfte. In diesem Artikel werden Daten des DEAS 2023 verwendet.

Gesundheit 65+ ist eine epidemiologische Längsschnittstudie zur gesundheitlichen Lage der Personen ab 65 Jahren in Deutschland. Die Basisbefragung wurde vom Robert Koch-Institut 2021/2022 auf Grundlage einer 2‑stufigen, geschichteten Zufallsstichprobe mit 3694 Personen ab 65 Jahren durchgeführt. Die Kontaktierung und Datenerhebung erfolgten nach einem Mixed-Mode-Design [30].

Für den vorliegenden Artikel werden Daten aus beiden Studien für hochaltrige Personen ab 80 Jahren, die in Privathaushalten leben, ausgewertet. Dabei geht es nicht um einen Vergleich der Datensätze, sondern um eine möglichst differenzierte, multidimensionale Beschreibung gesundheitlicher Ressourcen, Risiken und Defizite im hohen Alter.

### Indikatoren

Die genauen Frageformulierungen, Antwortkategorien und Operationalisierungen der Indikatoren sind im Onlinematerial zu diesem Artikel dargestellt.

Langfristige globale funktionale Einschränkungen werden mit dem *GALI* erfasst. Der Indikator wird als datensatzübergreifender Referenzindikator funktionaler Einschränkungen ausgewertet. Berichtet werden die Anteile der Hochaltrigen mit starken Einschränkungen. Bereichsspezifische Indikatoren körperlicher Funktionsfähigkeit und Frailty werden datensatzspezifisch ausgewertet: Mit dem DEAS 2023 werden Alltagsfunktionalität, Mobilitätseinschränkungen sowie Frailty und mit Gesundheit 65+ Seh- und Höreinschränkungen sowie Harninkontinenz analysiert.

Die *Alltagsfunktionalität* wird mit 10 Fragen aus der Subskala „Physical Functioning“ des SF-36 zur gesundheitsbezogenen Lebensqualität [9] erfasst. Die Befragten bewerteten auf einer 3‑stufigen Skala, inwieweit ihr aktueller Gesundheitszustand verschiedene Alltagstätigkeiten einschränkt (z. B. körperlich anstrengende oder mittelschwere Aktivitäten, Treppensteigen, Gehen, Bücken sowie Baden oder Anziehen). Die Antworten wurden pro Person summiert und auf eine Skala von 0 bis 100 transformiert. Höhere Werte dieses Summenscores zeigen geringere Einschränkungen und damit eine bessere funktionale Gesundheit an.

Zusätzlich werden zwei Einzelitems aus dieser Subskala zur Darstellung von *Mobilitätseinschränkungen* ausgewertet: „mehr als 1 km zu Fuß gehen“ und „einen Treppenabsatz steigen“. Für die Analysen wird für beide Items der Anteil der Hochaltrigen berichtet, die angegeben haben, dabei stark eingeschränkt zu sein.

*Seheinschränkungen* werden gemäß EHIS‑3 [31] mit der Frage erhoben, ob Schwierigkeiten beim Sehen bestehen, auch wenn eine Brille oder Kontaktlinsen getragen werden. Für die Analysen wird der Anteil der Personen ausgewiesen, die große Schwierigkeiten oder ein völliges Unvermögen beim Sehen angeben. *Höreinschränkungen* werden mit zwei Fragen erfasst, die Hörprobleme in Gesprächen mit einer anderen Person in einem ruhigen beziehungsweise lauteren Raum thematisieren – auch bei Nutzung eines Hörgeräts. Für die Analysen wird der Anteil der Personen ausgewiesen, die in mindestens einer dieser Situationen große Schwierigkeiten oder ein Unvermögen angeben.

Für die Analysen zur *Harninkontinenz* wird der Anteil der Personen ausgewiesen, die angeben, in den letzten 12 Monaten Harninkontinenz beziehungsweise Probleme mit der Blasenkontrolle gehabt zu haben.

Der im DEAS verwendete *Frailty-Index* wurde als adaptierte Proxy-Operationalisierung konstruiert. Er orientiert sich konzeptionell am Fried-Phänotyp [13], kann diesen jedoch aufgrund der im DEAS verfügbaren Informationen nicht vollständig abbilden. Einzelne Komponenten wie Greifkraft und Gehgeschwindigkeit wurden über Surrogatindikatoren operationalisiert. Der resultierende Frailty-Status ist daher nicht als direkte klinische Messung, sondern als surveybasierte Annäherung an körperliche Gebrechlichkeit zu verstehen. Der Frailty-Index nach Fried umfasst fünf Komponenten (Erfassung bzw. Surrogatindikator im DEAS): unbeabsichtigter Gewichtsverlust von mehr als 5 kg im letzten Jahr (Selbstangabe), Erschöpfung (CES-D-Skala; [32]), geringe körperliche Aktivität (seltener als einmal monatlich), eingeschränkte Mobilität (Nutzung einer Gehhilfe) sowie reduzierte Greifkraft (selbstberichtet als starke Einschränkung beim Heben oder Tragen von Einkaufstaschen; SF-36-PF-Skala; [9]). Der im DEAS verwendete Greifkraft-Indikator bildet nicht ausschließlich den Kraftaspekt ab, sondern kann auch durch Schmerzen, Gelenkerkrankungen und ähnliche Einschränkungen beeinflusst sein. Diese methodische Ungenauigkeit musste in Kauf genommen werden, da dieser Indikator die einzige Information im DEAS zu Muskelkraft ist. Aus der Summe der fünf Komponenten wurde ein Score gebildet, anhand dessen drei Gruppen unterschieden wurden: Für die Analysen wird der Anteil der Personen ausgewiesen, der „robust“ (0 Punkte), „pre-frail“ (1–2 Punkte) oder „frail“ (3–5 Punkte) ist.

Alle Indikatoren werden nach Geschlecht (Frauen, Männer) und Einkommen differenziert ausgewertet und dargestellt. Es werden drei Einkommensgruppen auf Basis des bedarfsgewichteten Pro-Kopf-Einkommens (Nettoäquivalenzeinkommen des Haushalts) unterschieden: armutsgefährdete Haushalte mit weniger als 60 % des Medianeinkommens, Haushalte mit mittlerem Einkommen (60–150 %) und Haushalte mit höherem Einkommen (über 150 % des Medianeinkommens).

Ausgewiesen werden die Prävalenzen aller betrachteten Indikatoren der körperlichen Funktionsfähigkeit für Hochaltrige (≥ 80 Jahre). Alle Schätzungen erfolgen gewichtet und unter Berücksichtigung des jeweiligen Surveydesigns (Clusterung und Stratifizierung). Konfidenzintervalle werden berichtet und Gruppenunterschiede deskriptiv anhand der Intervallüberlappung eingeordnet.

Insgesamt wurden die Daten von 938 Personen aus dem DEAS 2023 und 2028 Personen aus der Studie Gesundheit 65+ für die Datenauswertung herangezogen. Eine Übersicht über die beiden Stichproben findet sich in Tab. 1. Während im DEAS 2023 keine Proxy-Interviews durchgeführt wurden, konnten in der Studie Gesundheit 65+ körperlich oder kognitiv eingeschränkte Personen auch über die Befragung von stellvertretenden Personen an der Studie teilnehmen. Für die folgenden Analysen wurden auch die Angaben eingeschlossen, die über Dritte erfolgt sind.

**Tab. 1:** Stichprobenbeschreibung der ab 80-jährigen Teilnehmenden der Studien DEAS 2023 und Gesundheit 65+ (2021/2022). Gewichtete Anteile und ungewichtete Fallzahlen.

Merkmal	Ausprägung	DEAS 2023 (Gesamt *n* = 938) Anteil in % (Anzahl *n*)	Gesundheit 65+ (Gesamt *n* = 2028) Anteil in % (Anzahl *n*)
Alter	Durchschnittsalter (Jahre)	84,7 (938)	84,4 (2028)
Geschlecht	Frauen	59,4 (437)	60,3 (957)
	Männer	40,6 (501)	39,7 (1071)
Einkommen	Armutsgefährdet	14,9 (97)	28,3 (471)
	Mittlere Einkommen	64,8 (668)	63,3 (1306)
	Höhere Einkommen	20,3 (173)	8,4 (251)
Subjektive Gesundheit	Sehr gut/gut	35,1 (376)	37,0 (838)
	Mittel	45,6 (404)	48,4 (888)
	Sehr schlecht/schlecht	19,3 (158)	14,6 (273)
Beantwortung des Fragebogens	Selbst	–	84,0 (1768)
	Durch Stellvertretungen^a^ k. A.	–	14,5 (228)
	Angabe fehlt	–	1,5 (32)

^a^ Von den 228 stellvertretenden Interviewpersonen waren 73 die (Ehe‑)Partnerin bzw. der (Ehe‑)Partner, 125 die Tochter bzw. der Sohn, 27 andere Familienangehörige, 2 eine Freundin bzw. ein Freund und bei einer Person die gesetzliche Vertretung

## Ergebnisse

### Globale Funktionalität, Alltagsfunktionalität und Mobilität

Knapp jede vierte Person im Alter von 80 Jahren und älter ist – gemessen mit *GALI* – stark funktional eingeschränkt. Das zeigt sich übereinstimmend in beiden Datensätzen. Die Prävalenzen liegen im DEAS bei 22,8 % und in Gesundheit 65+ bei 24,6 % (Tab. 2). Der Anteil von Frauen mit starken funktionalen Einschränkungen liegt bei 25,8 % (DEAS) bzw. 27,1 % (Gesundheit 65+). Im Vergleich liegt der Anteil bei Männern bei 18,3 % (DEAS) bzw. bei 20,9 % (Gesundheit 65+). Der Geschlechterunterschied ist jedoch nicht statistisch bedeutsam. Hinsichtlich der Einkommenslage zeigt sich bei Gesundheit 65+ ein sozialer Gradient: Hochaltrige mit armutsgefährdendem Einkommen berichten signifikant häufiger starke funktionale Einschränkungen als Personen in der Gruppe mit höheren Einkommen. Die Einkommensunterschiede sind im DEAS statistisch nicht signifikant.

**Tab. 2:** Anteil der ab 80-Jährigen mit starken funktionalen Einschränkungen (GALI) insgesamt sowie nach Geschlecht und Einkommen. Gewichtete Anteile mit 95 %-Konfidenzintervallen (KI)

Merkmal	Ausprägung	DEAS 2023		Gesundheit 65+	
		Anteil in % (95 %-KI)	Anzahl (*n*)	Anteil in % (95 %-KI)	Anzahl (*n*)
*Insgesamt*		*22,8 (18,2–28,1)*	*919*	*24,6 (21,7–27,9)*	*424*
Geschlecht	Frauen	25,8 (19,6–33,3)	425	27,1 (23,3–31,3)	227
	Männer	18,3 (13,0–25,2)	494	20,9 (17,6–24,5)	197
Einkommen	Armutsgefährdet	24,9 (14,0–40,4)	95	30,5 (24,7–37,0)	115
	Mittlere Einkommen	22,6 (17,6–28,6)	662	23,2 (19,5–27,2)	270
	Höhere Einkommen	21,8 (12,4–35,4)	162	16,7 (11,3–24,1)	39

Der Mittelwert der *Alltagsfunktionalität*, gemessen mit dem SF-36-PF, liegt bei den 80-Jährigen und Älteren bei 58,1 (auf einer Skala von 0–100, wobei höhere Werte eine bessere körperliche Funktionsfähigkeit anzeigen; Tab. 3). Männer haben mit einem Mittelwert von 65,6 signifikant höhere Werte als Frauen (Mittelwert von 52,9), was auf eine bessere selbstberichtete körperliche Funktionsfähigkeit bei hochaltrigen Männern hinweist.

**Tab. 3:** Mittelwerte auf der Skala Physical Functioning (PF) des Short Form-36 Health Survey (SF-36) und Anteile starker Mobilitätseinschränkungen bei ab 80-Jährigen insgesamt sowie nach Geschlecht und Einkommen, DEAS 2023. Gewichtete Mittelwerte bzw. Anteile mit 95 %-Konfidenzintervallen (KI)

Merkmal	Ausprägung	SF-36-PF		Starke Mobilitätseinschränkungen			
				> 1 km zu Fuß gehen		1 Treppenabsatz steigen	
		Mittelwert (95 %-KI)	Anzahl (*n*)	% (95 %-KI)	Anzahl (*n*)	% (95 %-KI)	Anzahl (*n*)
*Insgesamt*		*58,1 (54,4–61,8)*	*925*	*30,3 (24,4–36,8)*	*934*	*16,9 (13,3–21,3)*	*937*
Geschlecht	Frauen	52,9 (48,0–57,9)	430	33,0 (24,9–42,3)	434	23,1 (17,8–29,4)	436
	Männer	65,6 (60,6–70,5)	495	26,3 (19,5–34,5)	500	7,9 (4,8–12,9)	501
Einkommen	Armutsgefährdet	50,8 (40,7–61,0)	96	34,6 (20,7–51,7)	96	26,3 (13,8–44,4)	97
	Mittlere Einkommen	59,8 (55,6–64,1)	659	30,6 (23,8–38,5)	666	13,3 (9,2–18,8)	667
	Höhere Einkommen	58,0 (48,9–67,0)	170	25,9 (15,7–39,7)	172	21,7 (12,2–35,4)	173

Bezogen auf die *Mobilität* berichtet rund ein Drittel der Hochaltrigen (30,3 %) starke Einschränkungen beim Gehen einer Strecke von mehr als einem Kilometer (Tab. 3). Frauen und Männer unterscheiden sich erneut nicht statistisch bedeutsam voneinander. Ein klarer sozialer Gradient zeigt sich nicht. Beim Steigen eines Treppenabsatzes sind 16,9 % der Hochaltrigen stark eingeschränkt. Frauen berichten fast dreimal so häufig starke Einschränkungen wie Männer (23,1 % vs. 7,9 %). Hinsichtlich des Einkommens ergibt sich kein konsistentes Muster.

### Sensorische Funktionalität

In der Altersgruppe der 80-Jährigen und Älteren berichten 10,4 % schwerwiegende Einschränkungen beim Sehen (Tab. 4). Frauen sind deutlich häufiger betroffen als Männer (12,7 % vs. 6,8 %) – der Unterschied ist statistisch signifikant. Zwischen den Einkommensgruppen gibt es keine statistisch bedeutsamen Unterschiede. Schwerwiegende Höreinschränkungen sind deutlich verbreiteter als entsprechende Seheinschränkungen: Mehr als ein Viertel der ab 80-Jährigen (27,4 %) berichtet entsprechende Beeinträchtigungen beim Hören (Tab. 4). Hier sind jedoch weder Geschlechts- noch Einkommensunterschiede statistisch signifikant.

**Tab. 4:** Anteil der ab 80-Jährigen mit starken Einschränkungen beim Sehen und Hören insgesamt, nach Geschlecht und Einkommen, Gesundheit 65+. Gewichtete Anteile mit 95 %-Konfidenzintervallen (KI)

Merkmal	Ausprägung	Starke Seheinschränkungen		Starke Höreinschränkungen	
		Anteil in % (95 %-KI)	Anzahl (*n*)	Anteil in % (95 %-KI)	Anzahl (*n*)
*Insgesamt*		*10,4 (8,7–12,4)*	*178*	*27,4 (24,7–30,3)*	*531*
Geschlecht	Frauen	12,7 (10,2–15,8)	113	26,8 (22,9–31,1)	242
	Männer	6,8 (4,9–9,5)	65	28,4 (25,2–31,7)	289
Einkommen	Armutsgefährdet	10,7 (7,8–14,7)	46	30,1 (25,2–35,5)	137
	Mittlere Einkommen	10,4 (8,5–12,8)	113	26,3 (22,9–29,9)	335
	Höhere Einkommen	8,9 (4,8–16,0)	19	27,2 (19,7–36,3)	59

### Kontinenzfunktion

40,3 % der 80-Jährigen und Älteren berichten über Probleme mit der Kontrolle der Blase bzw. Harninkontinenz in den letzten 12 Monaten (Tab. 5). Frauen sind signifikant häufiger von Harninkontinenz betroffen als Männer (44,8 % vs. 33,5 %). Zwischen den Einkommensgruppen gibt es Unterschiede in der Größenordnung von rund 5 Prozentpunkten (armutsgefährdet: 43,9 %; mittlere Einkommen: 38,8 %; höhere Einkommen: 39,9 %), die jedoch nicht statistisch signifikant sind.

**Tab. 5:** Anteil der ab 80-Jährigen mit Harninkontinenz in den letzten 12 Monaten insgesamt, nach Geschlecht und Einkommen, Gesundheit 65+. Gewichtete Anteile mit 95 %-Konfidenzintervallen (KI)

Merkmal	Ausprägung	Harninkontinenz	
		Anteil in % (95 %-KI)	Anzahl (*n*)
*Insgesamt*		*40,3 (37,3–43,5)*	*747*
Geschlecht	Frauen	44,8 (40,1–49,5)	413
	Männer	33,5 (30,1–37,2)	335
Einkommen	Armutsgefährdet	43,9 (38,4–49,6)	191
	Mittlere Einkommen	38,8 (34,7–43,0)	466
	Höhere Einkommen	39,9 (31,4–49,1)	90

Gebrechlichkeit (Frailty): Im DEAS 2023 zeigt knapp die Hälfte der 80-Jährigen und Älteren keine Anzeichen körperlicher Vulnerabilität im Sinne des DEAS-Frailty-Index (48,5 %; Tab. 6). Weitere 43,0 % sind als „pre-frail“ und 8,6 % als „frail“ einzustufen. Frailty tritt bei Frauen häufiger auf als bei Männern (12,1 % vs. 3,5 %), während Männer häufiger als robust gelten (59,5 % vs. 40,8 %); die Geschlechtsunterschiede sind jedoch nicht statistisch signifikant. Nach Einkommen zeigen sich beim Anteil frailer Personen keine konsistenten Unterschiede.

**Tab. 6:** Anteil der ab 80-Jährigen nach DEAS-Frailty-Stufen insgesamt, nach Geschlecht und Einkommen, DEAS 2023. Gewichtete Anteile mit 95 %-Konfidenzintervallen (KI)

Merkmal	Ausprägung	Frail		Pre-frail		Robust	
		Anteil in % (95 %-KI)	Anzahl (*n*)	Anteil in % (95 %-KI)	Anzahl (*n*)	Anteil in % (95 %-KI)	Anzahl (*n*)
*Insgesamt*		*8,6 (5,6–13,0)*	*38*	*43,0 (36,7–49,5)*	*310*	*48,5 (42,2–54,7)*	*458*
Geschlecht	Frauen	12,1 (7,4–19,2)	27	47,1 (38,4–56,0)	164	40,8 (32,3–49,9)	173
	Männer	3,5 (1,2–9,4)	11	37,0 (27,9–47,1)	146	59,5 (49,9–68,5)	285
Einkommen	Armutsgefährdet	10,0 (3,1–28,2)	3	62,1 (45,1–76,6)	46	27,9 (15,7–44,4)	31
	Mittlere Einkommen	7,3 (4,3–12,2)	28	41,1 (33,2–49,5)	212	51,6 (43,5–59,6)	343
	Höhere Einkommen	12,1 (4,3–29,4)	7	32,2 (21,1–45,6)	52	55,8 (41,4–69,3)	84

## Diskussion

### Globale und bereichsspezifische körperliche Funktionalität

Als Maß der globalen körperlichen Funktionalität wurde in beiden Datensätzen der *Global Activity Limitation Indicator (GALI)* eingesetzt [6, 7, 33]. Danach berichten etwa 3 Viertel der Hochaltrigen in Privathaushalten keine oder nur mittlere funktionale Einschränkungen, während rund ein Viertel stark eingeschränkt ist und damit in seiner selbstständigen Lebensführung gefährdet sein kann oder bereits Unterstützung benötigt. Dieses Muster zeigt sich konsistent in beiden Datensätzen. Frauen berichten in beiden Studien häufiger starke Einschränkungen als Männer, die Unterschiede sind jedoch nicht statistisch signifikant. Zugleich deutet sich ein sozialer Gradient an: Hochaltrige mit armutsgefährdendem Einkommen sind häufiger stark funktional eingeschränkt als Personen mit mittleren oder höheren Einkommen. In der Studie Gesundheit 65+ ist dieser Zusammenhang statistisch signifikant. Vergleichbare Befunde liegen auch aus anderen aktuellen deutschen Studien zu Hochaltrigen vor [34, 35] und werden zudem durch europäische Daten zu sozialen Ungleichheiten der funktionalen Gesundheit im höheren Lebensalter gestützt [36].

Dass Männer sowohl beim Summenscore des SF-36 höhere Werte der *Alltagsfunktionalität* erreichen als auch seltener starke Mobilitätseinschränkungen beim Treppensteigen berichten als Frauen, steht im Einklang mit internationalen Befunden, die auch für andere Indikatoren Geschlechterunterschiede zugunsten einer besseren Funktionalität der Männer zeigen. Dieses Muster kann als Hinweis auf das häufig beschriebene „male-female health-survival paradox“ interpretiert werden: Frauen haben zwar eine höhere Lebenserwartung, berichten jedoch häufiger gesundheitliche Einschränkungen und funktionale Beeinträchtigungen als Männer [20, 22, 37]. Daraus ergibt sich die Notwendigkeit geschlechtersensibler Präventions- und Versorgungsansätze, die insbesondere auf den Erhalt von Muskelkraft, Mobilität und funktionaler Selbstständigkeit bei Frauen im höheren Alter zielen.

### Sensorische Funktionalität

Sehen und Hören sind zentrale Voraussetzungen für Kommunikation, soziale Interaktion und Alltagsbewältigung. Entsprechend erhöhen sensorische Beeinträchtigungen das Risiko für Isolation, funktionale Einschränkungen und Unterstützungsbedarf [11, 38, 39]. Etwa 10 % der Hochaltrigen berichten starke Seh-, über ein Viertel starke Höreinschränkungen. Geschlechterunterschiede zeigen sich nur beim Sehen, mit höheren Anteilen bei Frauen. Ein erheblicher Anteil der Hochaltrigen ist demnach in der Teilhabe am gesellschaftlichen Leben durch sensorische Einschränkungen beeinträchtigt. Frühzeitige Prävention und Versorgung, etwa regelmäßige Screeninguntersuchungen und ein niedrigschwelliger Zugang zu Sehhilfen und Hörgeräten, können dem entgegenwirken.

### Kontinenzfunktion

Rund 40 % der hochaltrigen Menschen in Privathaushalten berichten Probleme mit Harninkontinenz; sie ist demnach ein erhebliches Gesundheitsproblem dieser Lebensphase. Auch hier sind Frauen häufiger betroffen als Männer; knapp jede zweite hochaltrige Frau berichtet entsprechende Beschwerden. Harninkontinenz kann die Lebensqualität stark beeinträchtigen und ist häufig mit Alltagseinschränkungen sowie mit Scham, Rückzug aus sozialen Aktivitäten und erhöhtem Unterstützungsbedarf verbunden [12]. Hier besteht demnach Bedarf an gezielter Prävention und Versorgung, aber auch an Information zur Entstigmatisierung des Themas. Ein sozialer Gradient der Harninkontinenz nach Einkommen zeigt sich in den vorliegenden Daten nicht.

### Gebrechlichkeit (Frailty)

Der Frailty-Ansatz ergänzt die betrachteten Einzelindikatoren um eine integrierte Perspektive auf altersbedingte Vulnerabilität und damit auf einen zentralen *Risikofaktor* eingeschränkter Funktionsfähigkeit. Die in diesem Beitrag verwendete adaptierte Proxy-Operationalisierung des Frailty-Phänotyps nach Fried (vgl. Methodenteil) ist nicht als klinische Frailty-Diagnostik zu verstehen, sondern als populationsbezogene Annäherung an körperliche Vulnerabilität und Gebrechlichkeit im hohen Alter. Der Indikator eignet sich insbesondere zur Beschreibung von Verteilungsmustern und Gruppenunterschieden innerhalb des DEAS.

Ein erheblicher Anteil der Hochaltrigen in Privathaushalten – etwa die Hälfte – zeigt nach der verwendeten adaptierten Fassung des Fried-Index keine oder nur geringe Anzeichen körperlicher Vulnerabilität, was hier als robuster Status bezeichnet wird. Knapp 9 % der Hochaltrigen können danach als „frail“ gelten. Da ausschließlich Personen in Privathaushalten berücksichtigt sind und gesundheitlich stark eingeschränkte Personen seltener an Surveys teilnehmen, dürfte dieser Frailty-Anteil unterschätzt sein. Frauen sind häufiger „frail“, Männer häufiger robust – das steht im Einklang mit internationalen Befunden, auch wenn dieser Befund im DEAS nicht signifikant ist [22, 23, 40]. Zusammenhänge zwischen sozioökonomischem Status und Frailty wurden international berichtet [25]. In den DEAS-Daten deutet sich ein sozialer Gradient zugunsten höherer Einkommen an, erreicht jedoch keine statistische Signifikanz. Die Befunde zu Frailty, insbesondere bei hochaltrigen Frauen, unterstreichen die Bedeutung frühzeitiger Identifikation dieses Risikofaktors. Es bedarf präventiver Interventionen, um Frailty im hohen Alter möglichst zu verzögern und die funktionalen Ressourcen zu erhalten.

## Stärken und Limitationen

Bei beiden zugrunde liegenden Studien handelt es sich um bevölkerungsrepräsentative Erhebungen, die Personen ab 80 Jahren in Privathaushalten einschließen. Durch altersgemäße Erhebungsinstrumente und Durchführung der Erhebung konnten valide Aussagen zum Gesundheitszustand der Hochaltrigen getroffen werden.

Im *DEAS 2023* stellt die vergleichsweise geringe Fallzahl hochaltriger Befragter eine zentrale Limitation dar. Aufgrund der kleinen Stichprobe erreichen auch deutlich erscheinende Unterschiede häufig keine statistische Signifikanz und sind daher mit Vorsicht zu interpretieren. Für 2026 ist eine gezielte Aufstockung dieser Altersgruppe geplant, wodurch die Aussagekraft künftig verbessert wird. Zudem basiert die Erhebung 2023 überwiegend auf einem Panel, das durch Attrition und Mortalität selektiver wird. Ein neuer Querschnittsgewichtungsfaktor wurde eingeführt, um dies auszugleichen [41]. Eine weitere Limitation besteht darin, dass die Frailty-Operationalisierung nur näherungsweise am Fried-Phänotyp orientiert ist und auf Surrogatindikatoren basiert. Dadurch ist die Vergleichbarkeit mit Studien eingeschränkt, die den Fried-Phänotyp vollständig oder klinisch erfassen.

Auch die Studie *Gesundheit 65+* weist Limitationen auf. Die COVID-19-Pandemie führte zu Verzögerungen, Anpassungen im Studiendesign und möglichen Verzerrungen der Stichprobe. Das Hausbesuchsdesign ermöglichte zwar die Einbeziehung gesundheitlich eingeschränkter Personen, war jedoch ressourcenintensiv und organisatorisch aufwendig. Zudem könnten erhöhte Datenschutzanforderungen und umfangreiche Öffentlichkeitsarbeit die Teilnahme beeinflusst haben [30].

Zu berücksichtigen ist außerdem, dass in beiden Datensätzen Personen in institutionellen Einrichtungen kaum vertreten und daher aus der Analyse ausgeschlossen worden sind. Stark gesundheitlich eingeschränkte Personen sind zudem schwerer zu erreichen. Die Ergebnisse beziehen sich somit auf die in Privathaushalten lebende Bevölkerung. Trotz dieser Einschränkungen liefern beide Datensätze wichtige bevölkerungsrepräsentative Hinweise zur funktionalen Gesundheit Hochaltriger in Deutschland.

## Fazit

Der zusammenfassende Blick auf die körperliche Funktionalität Hochaltriger zeigt, dass nur etwa ein Viertel der in Privathaushalten lebenden ab 80-Jährigen stark funktional eingeschränkt ist, während rund drei Viertel ohne oder nur mit mäßigen Einschränkungen leben. Ein erheblicher Teil der Hochaltrigen weist nur geringe Zeichen von Vulnerabilität auf und verfügt damit über vergleichsweise gute funktionale Ressourcen sowie eine höhere Belastbarkeit gegenüber gesundheitlichen Stressoren. Dies geht mit größerer Selbstständigkeit, sozialer Teilhabe und geringerer Inanspruchnahme von Gesundheits- und Pflegeleistungen einher – eine positive Entwicklung vor dem Hintergrund der demografischen Alterung. Gleichzeitig ist dieser Zustand nicht stabil: Auch robuste Hochaltrige können infolge akuter Ereignisse oder kumulativer Belastungen gebrechlich werden und an Selbstständigkeit verlieren. Daraus ergibt sich die Notwendigkeit, präventive Ansätze frühzeitig zu implementieren und Versorgungsstrukturen stärker an den spezifischen Bedarfen hochaltriger Menschen auszurichten, um funktionale Fähigkeiten möglichst lange zu erhalten.

Dies gilt in besonderem Maße für hochaltrige Frauen. In vielen untersuchten Dimensionen zeigen sie ungünstigere Werte als Männer, etwa hinsichtlich funktionaler Beeinträchtigungen im Alltag, sensorischer Einschränkungen und Frailty. Diese Unterschiede sind vor dem Hintergrund der höheren Lebenserwartung von Frauen zu interpretieren, die mit einer längeren Phase gesundheitlicher Einschränkungen einhergehen kann. Die Ergebnisse unterstreichen daher die Notwendigkeit geschlechtersensibler Präventions- und Versorgungsstrategien für das hohe Alter, die die erhöhte Vulnerabilität hochaltriger Frauen adressieren und ihre funktionale Gesundheit gezielt fördern.

Auch zwischen den Einkommensgruppen zeigen sich über mehrere Indikatoren hinweg tendenzielle Unterschiede in der körperlichen Funktionsfähigkeit, die jedoch statistisch nicht signifikant sind. Vor dem Hintergrund der begrenzten Fallzahlen sind diese Befunde vorsichtig zu interpretieren. Gleichwohl weisen die konsistenten Gradienten darauf hin, dass sozioökonomische Ungleichheiten auch im hohen Alter fortbestehen könnten.

## Supplementary Information

Onlinematerial: Frageformulierungen und Indikatorbildung

## Data Availability

Die DEAS-Daten stehen der wissenschaftlichen Forschung als Scientific Use Files (SUF) über das Forschungsdatenzentrum (FDZ) des Deutschen Zentrums für Altersfragen (DZA) kostenfrei zur Verfügung (www.fdz-dza.de). Die während der vorliegenden Studie Gesundheit 65+ erzeugten und/oder analysierten Datensätze sind nicht öffentlich zugänglich, da die Einwilligung (Informed Consent) der Studienteilnehmenden die öffentliche Bereitstellung der Daten nicht abdeckt, können aber auf begründete Anfrage beim FDZ des Robert Koch-Instituts (RKI) angefordert werden.
