# Supplementary material for: Physical functioning and frailty in very old age in Germany
Source: Bundesgesundheitsblatt Gesundheitsforschung Gesundheitsschutz. 2026 Aug 11;69(9):991–9. [Article in German] doi: 10.1007/s00103-026-04280-0 (PMC13562169; doi:10.1007/s00103-026-04280-0)
Supplement: Supplementary file 1 [file 103_2026_4280_MOESM1_ESM.pdf]

Onlinematerial zum Artikel „Körperliche Funktionsfähigkeit und Gebrechlichkeit (Frailty) im hohen Alter in Deutschland“: Frageformulierungen, Antwortkategorien und Bildung der Indikatoren

Indikator und Studie	Frageformulierung und Antwortkategorien	Bildung des Indikators
**GALI** (Global Activity Limitation Indicator)<br>DEAS 2023 und Gesundheit 65+	**DEAS 2023: Waren Sie während der letzten 6 Monate oder länger bei Dingen, die man üblicherweise so tut, aus gesundheitlichen Gründen eingeschränkt?**<br>1: Ja, stark eingeschränkt<br>2: Ja, eingeschränkt<br>3: Nein, nicht eingeschränkt<br><br>**Gesundheit 65+: Sind Sie dauerhaft durch ein gesundheitliches Problem bei Tätigkeiten des normalen Alltagslebens eingeschränkt?**<br>Würden Sie sagen, Sie sind...<br>- stark eingeschränkt?<br>- mäßig eingeschränkt?<br>- nicht eingeschränkt?	Prozentuale Anteile<br>„Ja, stark eingeschränkt“ bei DEAS 2023 sowie „stark eingeschränkt“ bei Gesundheit 65+
**Alltagsfunktionalität, Summenscore des PF (Physical Functioning)-Moduls des SF-36**<br>DEAS 2023	**Sind Sie durch Ihren derzeitigen Gesundheitszustand bei diesen Tätigkeiten stark eingeschränkt, etwas eingeschränkt oder überhaupt nicht eingeschränkt?**<br>A: Anstrengende Tätigkeit, z.B. schnell laufen, schwere Gegenstände heben, anstrengenden Sport treiben<br>B: Mittelschwere Tätigkeiten, z.B. einen Tisch verschieben, staubsaugen, kegeln, Golf spielen<br>C: Einkaufstaschen heben oder tragen<br>D: Mehrere Treppenabsätze steigen<br>E: Einen Treppenabsatz steigen<br>F: Sich beugen, knien, bücken<br>G: Mehr als 1 Kilometer zu Fuß gehen<br>H: Mehrere Straßenkreuzungen weit zu Fuß gehen<br>I: Eine Straßenkreuzung weit zu Fuß gehen<br>K: Sich baden oder anziehen<br><br>1: Ja, stark eingeschränkt<br>2: Ja, eingeschränkt<br>3: Nein, nicht eingeschränkt	Die Berechnung des Scores für die körperliche Funktionsfähigkeit (PF-Modul des SF-36) erfolgt in zwei Schritten aus den zehn entsprechenden Items A bis K. Zuerst werden die dreistufigen Antwortkategorien in standardisierte Punktwerte transformiert: Eine starke Einschränkung erhält 0 Punkte, eine mäßige Einschränkung 50 Punkte und keine Einschränkung 100 Punkte. Danach wird für jede Person der Mittelwert aus allen gültig beantworteten Items berechnet. Der finale Score liegt somit standardisiert zwischen 0 (maximale Einschränkung) und 100 (vollständige Freiheit von Einschränkungen)<br>Berichtet werden die Mittelwerte für die jeweilige Gruppe.

**Mobilitätsein-schränkungen**, **SF-36**<br>DEAS 2023	**Sind Sie durch Ihren derzeitigen Gesundheitszustand bei diesen Tätigkeiten stark eingeschränkt, etwas eingeschränkt oder überhaupt nicht eingeschränkt?**<br>G: Mehr als 1 Kilometer zu Fuß gehen<br>E: Einen Treppenabsatz steigen<br><br>1: stark eingeschränkt<br>2: etwas eingeschränkt<br>3: überhaupt nicht eingeschränkt	Prozentuale Anteile „stark eingeschränkt“
**Seheinschränkungen**<br>Gesundheit 65+	**Haben Sie Schwierigkeiten beim Sehen, selbst wenn Sie eine Brille oder Kontaktlinsen tragen?**<br>- Keine Schwierigkeiten<br>- Einige Schwierigkeiten<br>- Große Schwierigkeiten<br>- Es ist mir gar nicht möglich / Ich bin dazu nicht in der Lage.	Summe der prozentualen Anteile „große Schwierigkeiten“ und „Es ist mir gar nicht möglich / Ich bin dazu nicht in der Lage.“
**Hören**<br>Gesundheit 65+	**Haben Sie Schwierigkeiten zu hören, was in einem Gespräch mit einer anderen Person**<br>**… in einem ruhigen Raum gesagt wird, selbst wenn Sie ein Hörgerät tragen?**<br><br>**… in einem lauteren Raum gesagt wird, selbst wenn Sie ein Hörgerät tragen?**<br><br>- Keine Schwierigkeiten<br>- Einige Schwierigkeiten<br>- Große Schwierigkeiten<br>- Es ist mir gar nicht möglich / Ich bin dazu nicht in der Lage.	Summe der prozentualen Anteile in mindesten einer der beiden Situationen „große Schwierigkeiten“ und „Es ist mir gar nicht möglich / Ich bin dazu nicht in der Lage.“
**Harninkontinenz**<br>Gesundheit 65+	**Hatten Sie in den letzten 12 Monaten Harninkontinenz bzw. Probleme, die Blase zu kontrollieren?**<br>- Ja.<br>- Nein.	Prozentuale Anteile „ja“

**DEAS-Frailty-Index**<br>DEAS 2023	**Unbeabsichtigter Gewichtsverlust:**<br>Haben Sie im letzten Jahr mehr als 5 Kilogramm abgenommen?<br><br>1: Ja<br>2: Nein<br><br>Geschah dies beabsichtigt z. B. durch Diät, körperliche Aktivität – oder unbeabsichtigt?<br><br>1: Beabsichtigt<br>2: Unbeabsichtigt<br>⇨ Kriterium erfüllt, wenn mehr als 5 kg unbeabsichtigt abgenommen = 1 Punkt im Frailty-Score<br><br>**Erschöpfung (CES-D-Skala):**<br>Bitte nennen Sie mir für jede der Aussagen, die ich nun vorlese, die Antwort, die Ihrem Befinden während der letzten Woche am besten entspricht.<br>Während der letzten Woche ...<br>D: war ich deprimiert / niedergeschlagen<br>P: konnte ich mich zu nichts aufraffen<br>1: Selten oder überhaupt nicht, also weniger als 1 Tag<br>2: Manchmal, also an 1 bis 2 Tagen<br>3: Öfters, also an 3 bis 4 Tagen<br>4: Meistens oder die ganze Zeit, also an 5 bis 7 Tagen<br>⇨ Kriterium erfüllt, wenn für D und P Antwort 4 (meistens oder die ganze Zeit) angegeben wurde = 1 Punkt im Frailty-Score<br><br>**Geringe körperliche Aktivität:**<br>Wie oft treiben Sie <u>anstrengende körperliche Aktivitäten</u>? Gemeint sind Aktivitäten, die starke körperliche Anstrengungen erfordern und bei denen Sie deutlich stärker atmen als normal, wie z. B. Tragen schwerer Lasten, anstrengende Gartenarbeiten, Aerobic oder schnelles Fahrradfahren. Bitte denken Sie an alle anstrengenden körperlichen Aktivitäten, also jene im Rahmen Ihrer Arbeit, in Haus(halt) und Garten, um von einem Ort zum anderen zu kommen und in Ihrer Freizeit.<br><br>Wie oft treiben Sie <u>moderate körperliche Aktivitäten</u>? Gemeint sind Aktivitäten mit mäßiger körperlicher Anstrengung bei denen Sie ein wenig stärker atmen als normal, wie z. B. Tragen leichter Lasten, leichte Gartenarbeiten, Wandern, Schwimmen oder Fahrradfahren bei gewöhnlicher Geschwindigkeit. Bitte denken Sie an alle moderaten körperlichen Aktivitäten, also	**Konstrukt aus den DEAS-Daten in Anlehnung an Fried et al. 2001** aus den Kriterien:<br>unbeabsichtigter Gewichtsverlust von mehr als 5 kg im letzten Jahr (Selbstangabe),<br>Erschöpfung (CES-D-Skala)<br>geringe körperliche Aktivität (seltener als einmal monatlich),<br>eingeschränkte Mobilität (Nutzung einer Gehhilfe) sowie<br>reduzierte Greifkraft (selbstberichtet als starke Einschränkung beim Heben oder Tragen von Einkaufstaschen; SF-36-PF-Skala)<br><br>Aus der Summe der fünf Komponenten wurde ein Score gebildet, anhand dessen drei Gruppen unterschieden wurden: Für die Analysen wird der Anteil der Personen ausgewiesen, der „robust“ (0 Punkte), „pre-frail“ (1–2 Punkte) oder „frail“ (3–5 Punkte) ist.

	jene im Rahmen Ihrer Arbeit, in Haus(halt) und Garten, um von einem Ort zum anderen zu kommen und in Ihrer Freizeit. Beziehen Sie Spaziergänge bitte nicht mit ein.<br><br>Wie oft treiben Sie leichte körperliche Aktivitäten? Gemeint sind Aktivitäten, die mit geringem körperlichem Einsatz verbunden sind und bei denen Sie nicht stärker atmen als normal, wie z. B. zu Hause umhergehen, Gänge von einem Ort zum anderen sowie Spaziergänge, die Sie zur Entspannung, als Sport, zur Übung oder aus Muße unternommen haben.<br><br>- Täglich,<br>- Mehrmals in der Woche,<br>- Einmal in der Woche,<br>- Ein- bis dreimal im Monat,<br>- Seltener,<br>- Nie<br>⇨ Kriterium erfüllt, wenn in allen drei Abstufungen körperlicher Aktivität jeweils „Seltener“ oder „Nie“ angegeben wurde = 1 Punkt im Frailty-Score<br><br>**Langsame Gehgeschwindigkeit:** operationalisiert als eingeschränkte Mobilität<br>Benutzen Sie eines oder mehrere der folgenden Hilfsmittel?<br>- Stock / Gehhilfe<br>- Gehwagen, Rollator, Delta-Rad<br>- Rollstuhl<br>⇨ Kriterium erfüllt, wenn mind. eins dieser Hilfsmittel genutzt wird = 1 Punkt im Frailty-Score<br><br>**Reduzierte Greifkraft:** operationalisiert als starke Einschränkung beim Heben oder Tragen von Einkaufstaschen; SF-36-PF-Skala<br>Sind Sie durch Ihren derzeitigen Gesundheitszustand bei diesen Tätigkeiten stark eingeschränkt, etwas eingeschränkt oder überhaupt nicht eingeschränkt?<br>C: Einkaufstaschen heben oder tragen<br><br>1: Ja, stark eingeschränkt<br>2: Ja, eingeschränkt<br>3: Nein, nicht eingeschränkt<br>⇨ Kriterium erfüllt, wenn starke Einschränkung berichtet wird = 1 Punkt im Frailty-Score	
